# Associations of social disconnection risk and activities of daily living among older adults

**DOI:** 10.1093/geroni/igaf122.3715

**Published:** 2025-12-31

**Authors:** Amy Neal, Matthew Smith

**Affiliations:** Texas A&M University, College Station, Texas, United States; Texas A&M University, College Station, Texas, United States

## Abstract

Age-related changes such as sarcopenia, certain chronic conditions, and physical inactivity can cause older adults to become frail. Frailty can make activities of daily living (ADLs) difficult and increase the risk of falling. While mobility impairments may diminish social connection among older adults, there is limited documentation of this relationship. The purpose of this study was to examine the relationship between social connection and difficulty performing three mobility-based ADLs (i.e., walking/climbing stairs, dressing/bathing, doing errands). A sample of 3,602 adults aged 65+ from an internet-delivered survey were analyzed using multinomial logistic regression. Participants were grouped into three groups: no ADL difficulties (71.6%), 1 ADL difficulty (21.3%), and 2+ ADL difficulties (7.1%). The primary predictor variable was social disconnection risk measured using the Upstream Social Interaction Risk Scale (U-SIRS-13). On average, participants were age 70.57(±4.76) and had 3.29(2.52) self-reported chronic conditions. About 16% of the sample reported falling one or more times in the last 12 months. Compared to participants with no ADL difficulties, participants with more social disconnection risk (p < 0.001), more self-reported chronic conditions (p < 0.001), and who fell one or more times in the past year (p < 0.001) had significantly higher odds of reporting 1 ADL difficulty and 2+ ADL difficulties, respectively. Results suggest a that older adults with more mobility impairments may feel more socially disconnected and have more complex disease profiles. Findings support that older adults may benefit from evidence-based fall prevention programming, which can alleviate fall-related risk factors, while improving physical function and promoting social connection with others.

